# Magnesium: Hype and Health Benefits

**DOI:** 10.1007/s13668-026-00786-w

**Published:** 2026-07-22

**Authors:** Senthilkumar Sankararaman, Zoe Memel, Jason Dubroff, Jason P. Rocha

**Affiliations:** 1 Department of Pediatric Gastroenterology, Cleveland Clinic Children’s Hospital, Cleveland, OH USA; 2 Department of Gastroenterology, Sutter Medical Group of the Redwoods, Sutter Health, Santa Rosa, CA USA; 3 Department of Gastroenterology, Hepatology, and Nutrition, University of Utah School of Medicine, Salt Lake City, UT USA; 4 Department of Medicine, Division of Gastroenterology and Nutrition, University of Texas Health San Antonio, San Antonio, TX USA

**Keywords:** Magnesium, Hypomagnesemia, Supplements, Calcium

## Abstract

**Purpose of Review:**

Magnesium plays an indispensable role in human health and is intricately involved in many crucial physiological and metabolic pathways. True biochemical magnesium deficiency is rarely encountered in clinical practice but subclinical and functional deficiency of magnesium appears to be more common and is reported in many chronic health disorders.

**Recent Findings:**

Estimating magnesium status is challenging and best evaluated by a combination of serum magnesium, urinary magnesium, and dietary magnesium intake. Many randomized controlled trials (RCTs) have demonstrated the efficacy of magnesium supplementation in various chronic disorders such as constipation, dyspepsia, mood disorders, metabolic diseases, and bone health. However, magnesium supplements are heavily advertised for several purported and hyped benefits, which require more high-quality evidence to support these claims. Caution should be exerted when supplementing magnesium to people who are predisposed to adverse effects, such as in renal insufficiency.

**Summary:**

Studies noted the significant role of magnesium in the acute management of eclampsia, asthma, and migraine. RCTs have noted a significant role of magnesium supplements in many chronic disorders, such as constipation, gastroesophageal reflux disease, metabolic dysfunction (diabetes, hypertension, dyslipidemia, and obesity), and mood disorders.

## Introduction

Magnesium is the second most prevalent intracellular cation (after potassium) and overall fourth most abundant cation in the body (after sodium, potassium, and calcium) [1]. Magnesium is a cofactor in over 300 enzymatic reactions that regulate a wide variety of indispensable biochemical reactions, including energy production (ATP synthesis via oxidative phosphorylation and glycolysis), protein synthesis, and bone health [2, 3]. Magnesium has an indispensable role in carbohydrate metabolism, via its role in glucose and insulin metabolism [4]. Magnesium is required for DNA and RNA synthesis and also plays an essential role in the transport of calcium and potassium ions across cell membranes, thereby regulating neuronal and muscular function [5]. It acts as a physiological antagonist to calcium in neuromuscular excitability and cardiac homeostasis [3, 4, 6]. Magnesium also plays an important role in maintaining intracellular cationic gradients, keeping sodium and calcium low and potassium high [3, 5]. Hypomagnesemia has been associated with refractory hypokalemia, hypocalcemia, and metabolic alkalosis, and these disturbances improve only with optimization of magnesium status. Further, magnesium plays an important role in bone metabolism, including osteoblast proliferation and regulation of parathyroid hormone [7].

## Magnesium Status and Deficiency

In adults, roughly 25 g of magnesium can be found. At least half of this is present in bones, one-third in muscles, and approximately only 1% exists in the serum. Even though serum magnesium is the most commonly used pragmatic test for evaluating magnesium status, and normal magnesium levels do not exclude insufficiency [8, 9]. Low serum magnesium is noted only in severe stages of magnesium deficiency. Experts recommend a combination of serum magnesium, urinary magnesium, and dietary magnesium intake as the best way to determine magnesium status compared to using serum levels alone [10].

True magnesium deficiency can arise from poor dietary intake, patients with malnutrition (particularly during refeeding syndrome), mucosal intestinal disorders (intestinal malabsorption or excess intestinal loss), intestinal failure, chronic renal disease resulting in magnesium wasting, chronic alcoholism, and due to drug side effects and/or from drug interactions [3, 4, 10]. A number of medications interact with magnesium excretion. For example, diuretics (furosemide and hydrochlorothiazide), aminoglycosides, cisplatin, calcineurin inhibitors, and pentamidine increase the magnesium loss in the urine, predisposing to hypomagnesemia [11]. Similarly, magnesium loss is higher in patients with diabetes and chronic alcohol usage, predisposing to lower magnesium status [11, 12].

Also, the normal serum magnesium level is generally described as 1.7–2.4 mg/dL, and symptoms of hypomagnesemia include non-specific symptoms such as fatigue, lethargy, tiredness, and may progress to muscle cramps, tremors, and cardiac arrhythmias [5, 13]. Experts also classified magnesium status as follows: symptomatic hypomagnesemia (< 1.22 mg/dL), asymptomatic hypomagnesemia (1.22–1.81 mg/dL), chronic latent magnesium deficiency (1.82–2.05 mg/dL), normal range (2.06–2.33 mg/dL) [13]. Even though overt symptomatic biochemical magnesium deficiency is rarely encountered in day-to-day practice, subclinical and functional deficiency appear to be common and are reported in many chronic conditions.

National Health and Nutrition Examination Survey (NHANES) studies have noted that more than 50% of adults in the U.S. consume less magnesium than the recommended dietary allowance (RDA), and this trend did not change between 2003 and 2018 [14, 15]. Lower magnesium content in many commonly consumed foods, such as fruits and vegetables, is likely one of the main reasons for magnesium deficiency [16]. Experts have proposed a lower level of 2 mg/dL as a lower standard reference value to overcome chronic latent magnesium deficiency in several non-communicable diseases such as metabolic dysfunction, migraine, and mood disorders [5, 13]. Hence, magnesium supplementation is utilized in many chronic health issues, and also the manufacturers often claim purported health benefits as well.

## Dietary Sources, Formulations, and Pharmacokinetics

**Fig. 1:**
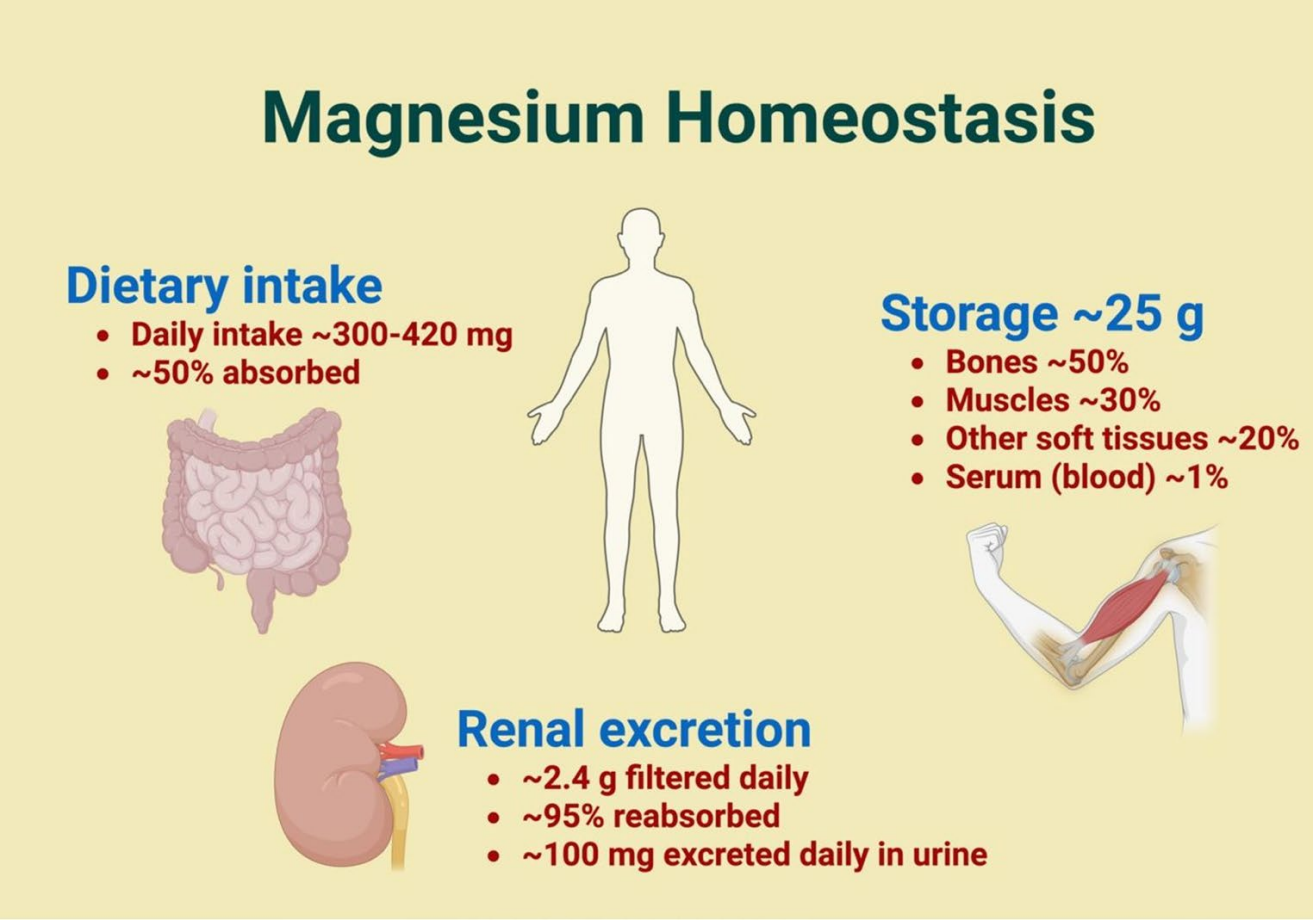
Magnesium homeostasis in the human body. Created with BioRender^(R)^

Magnesium is available from several sources, ranging from naturally occurring foods to magnesium-fortified products. Rich sources of magnesium include green leafy vegetables, wholegrains, nuts, and seeds [4, 17]. Fiber, phytates, and oxalates can hinder absorption. The primary site of absorption is the small bowel, particularly via the paracellular pathway, and nearly one-third (30–40%) of dietary magnesium (~ 100 mg) is absorbed, and the rest is eliminated via the stools [8, 9]. Nearly one-third is bound to albumin and approximately 60–70% as ionic form and about 10% as magnesium complexes in the serum [9, 11]. Magnesium homeostasis is tightly controlled by intestinal absorption, storage mainly in musculoskeletal tissues, and excretion by the kidneys (Fig. 1). About 100 mg of magnesium is excreted on a daily basis. As the main route of excretion is the kidneys, in people with renal insufficiency, magnesium supplementation should be carefully used to avoid hypermagnesemia. In food supplements, organic formulations appear to be better absorbed than inorganic salts. The (RDAs) for magnesium vary with age and sex as noted in Table 1.

**Table 1:** The recommended dietary allowances (RDAs) for magnesium in milligrams (mg) [17]

Age	Both sexes	
< 6 mon	30	
7–12 mon	75	
1–3 yrs	80	
4–8 yrs	130	
9–13 yrs	240	
	**Men**	Women*
14–18 yrs	410	360
19–30 yrs	400	310
> 30 yrs	420	320

(mon – months; yrs – years. *During pregnancy, the daily RDA may increase by at least 40 mg daily, and no major changes in requirement during lactation have been noted)

Supplemental magnesium is formulated in various forms, including salts (magnesium hydroxide, magnesium oxide) and chelates (e.g., magnesium glycinate) [18] (Table 2). Organic formulations such as citrate and glycinate are better tolerated and absorbed than inorganic salts such as oxide and chloride [8, 10]. Manufacturers often promote these different formulations with claims of superior absorption or organ-specific effects. For example, magnesium L-threonate is reported to cross the blood–brain barrier and is often marketed as promoting healthy brain function [19]. Similarly, magnesium malate is branded as the best magnesium supplement for energy and muscle support, given its involvement in the Krebs’ cycle and energy production. Magnesium is also marketed along with other dietary supplements and micronutrients such as vitamin D, calcium, and zinc. The upper intake level for magnesium from supplemental products is 350 mg for adults, and for the pediatric population, it ranges from 65 to 350 mg (based on the age of children and adolescents) [8]. Diarrhea or gastrointestinal distress is the most commonly reported adverse effect, specifically at high oral doses of magnesium [9]. Very large doses of magnesium supplements can lead to hypermagnesemia, and its manifestations include nausea, vomiting, paralytic ileus, urinary retention, and culminating in severe cases, life-threatening manifestations, such as extreme muscle weakness leading to dyspnea, profound hypotension, cardiac arrhythmias, and cardiorespiratory arrest [8]. As systemic magnesium is predominantly eliminated via the kidneys, administration of supplements in patients with renal insufficiency should be carefully considered to prevent these complications of hypermagnesemia.

**Table 2:** Some of the commonly available products of magnesium salts over the counter [4]

Compounds	Formulations	Comments
Magnesium hydroxide	• Solution, referred to as milk of magnesia (400 mg/5 mL), chewable tablets (400 mg)	• Used commonly as a laxative • Also used as an antacid (along with calcium salts) • Poorly absorbed
Magnesium oxide	• Tablet/caplet (180 mg, 250 mg, 500 mg), softgel (400 mg), gummy (200 mg, 400 mg), capsule (200 mg, 500 mg)	• Used as a laxative, nerve and muscle support, has poor bioavailability
Magnesium citrate	• Solution (290 mg/30 mL), softgel (250 mg), tablet (100 mg), chewable (85 mg)	• Solution is commonly used for bowel preparation and constipation • Also marketed for enhancing nerve, muscle, bone, and heart support • Better absorbed than oxide
Magnesium glycinate	• Gummy (100 mg, 200 mg) Capsule (100 mg,120 mg,400 mg, 500 mg), tablet (150 mg)	• Marketed for enhancing nerve, muscle, bone, and heart support, energy, calmness/mind relaxation

**Fig. 2:**
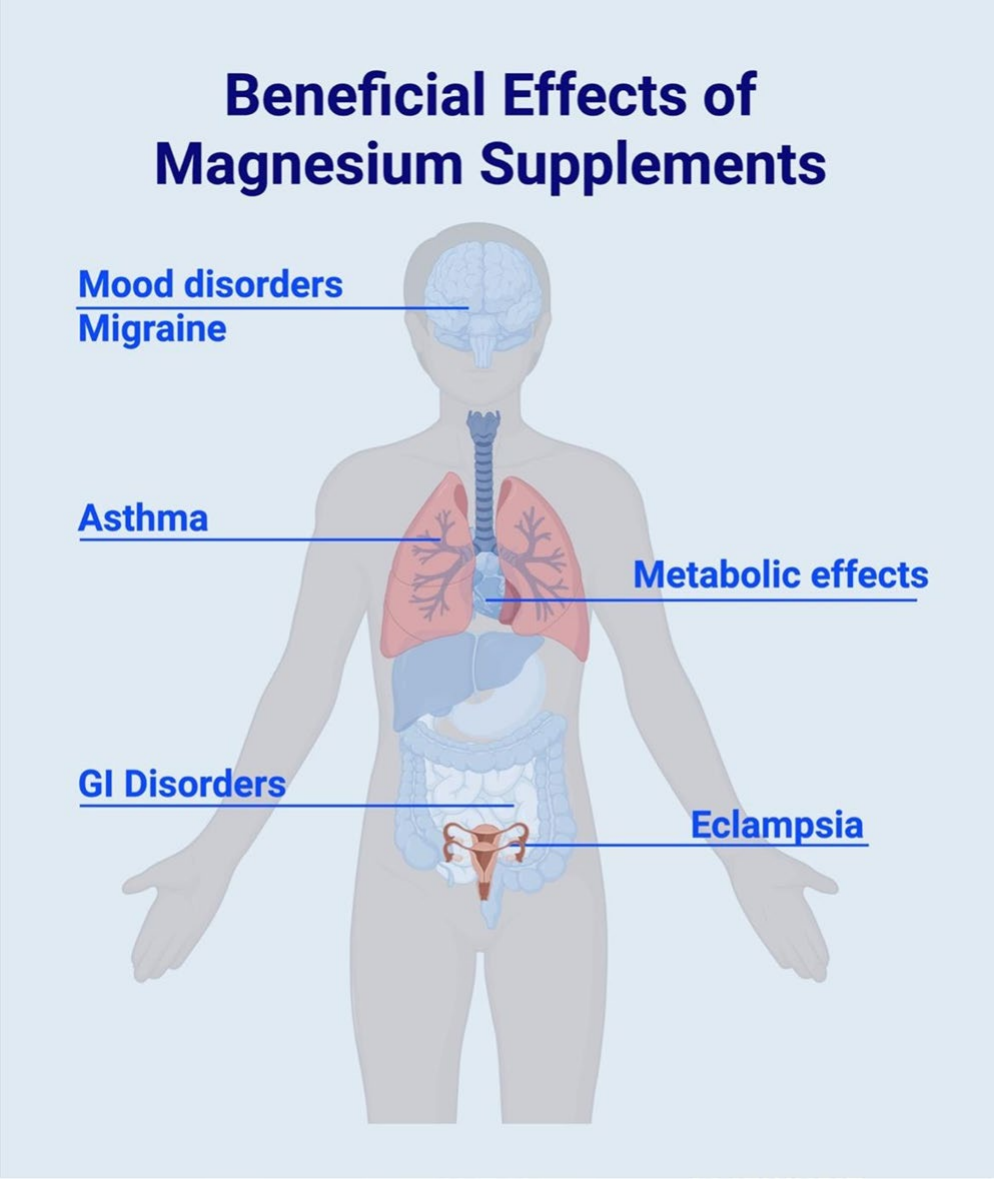
Beneficial effects of magnesium on various systems and organs. BioRender^(R)^

The 1994 Drug Supplement Health Education Act allows manufacturers to cite functional claims if they describe the role of a nutrient or dietary ingredient in affecting normal bodily function. For example, “magnesium promotes bone health.” Such claims must be accompanied by disclaimers stating that supplements are not intended to “diagnose, treat, cure or prevent any disease.” These marketing regulations, combined with magnesium’s involvement in numerous physiological processes, allow for opportunistic and potentially inflated health claims. Studies have noted that intravenous magnesium sulfate has been noted to be very effective in the management of many emergencies, such as eclampsia, torsades de pointes, acute severe asthma, and acute migraine which is discussed elsewhere [16, 20–24]. In the current times, several magnesium supplements are available on the market, either alone or in combination with other nutrients/ingredients, and are being marketed for several purposes. The global magnesium market is steadily growing, and in the year 2025, it reached ~ 53 million USD and is currently projected at an annual growth rate of ~ 5%, and reach at 81.4 million USD in 2033 [25]. Similarly, the popularity of magnesium among researchers has been increasing steadily, and a search in PubMed^®^ noted 1789 articles in the year 1995 and 4658 in 2025 [26]. In this article, we evaluated both evidence-based claims and also reviewed the purported health benefits of magnesium on various systems and disorders (Fig. 2).

### Gastrointestinal Benefits

Different magnesium salts are being used for various clinical effects. Poorly absorbed salts such as magnesium citrate, magnesium oxide, and magnesium hydroxide can serve as an osmotic laxative at higher doses, drawing water into the intestinal lumen and stimulating peristalsis. Magnesium citrate is the fastest-acting agent and is often used in combination with stimulant laxatives for rapid bowel preparations (for colonoscopic procedures) and for immediate relief of acute constipation [27, 28]. Magnesium oxide has the strongest evidence for the management of chronic constipation. The most recent clinical practice guideline [29] on the pharmacological management of chronic idiopathic constipation supports magnesium oxide as an effective management strategy for chronic idiopathic constipation, citing two, placebo-controlled randomized control trials (RCTs) using 1.5 g of magnesium oxide daily for 4 weeks in patients with chronic constipation with a resulting higher improvement in constipation in the treatment arm compared with placebo (RR 3.92, 95% CI 2.04–7.56) [30, 31]. This guideline advises caution on magnesium use as a laxative in patients with renal insufficiency or pregnancy. Magnesium hydroxide, commonly known as milk of magnesia, is a widely used over-the-counter agent for chronic constipation, although high-quality clinical trial data supporting its use are limited. Adherence to this laxative could be challenging due to its unpleasant taste.

Magnesium salts, primarily magnesium hydroxide, carbonate, and trisilicate, are also utilized for heartburn relief and functional dyspepsia and are often combined with aluminum hydroxide to reduce diarrhea and optimize acid neutralization. These agents neutralize gastric hydrochloric acid to form magnesium chloride, increase gastric pH, and provide rapid relief of acid-mediated symptoms. Few studies have examined magnesium salts alone for acid reflux, but several RCTs support magnesium-aluminum combination agents for heartburn relief. A 2017 RCT comparing magnesium-aluminum antacid gel (magnesium hydroxide and aluminum hydroxide; Maalox^®^) with an alginate reflux suppressant (liquid Gaviscon^®^) in 100 pregnant women found high rate of ≥ 50% reduction in heartburn frequency and intensity in both cohorts, with no statistically or clinically significant differences (RR for ≥ 50% reduction in frequency ≈ 1.08, 95% CI 0.75–1.55) [32]. Alginate-based formulations, which may include magnesium salts to enhance acid suppression, have also demonstrated safety and efficacy in pediatric populations [33]. These agents are useful for as-needed symptom relief, particularly when proton pump inhibitors or histamine blockers are being avoided. However, magnesium-based therapies are not effective for managing erosive esophagitis or for chronic daily therapy due to the short duration of action and potential for worsening rebound symptoms.

### Neuropsychiatric Benefits (for mood disorders and migraine)

Magnesium insufficiency/deficiency has been hypothesized to contribute to mood disorders through effects on hippocampal synapses and its role as a natural N-methyl-D-aspartate (NMDA) receptor antagonist [34]. Several small RCTs have also demonstrated that magnesium supplementation for as little as six weeks improved symptoms of depression and anxiety. In a RCT trial by Tarleton et al., provision of 248 mg of elemental magnesium daily for 6 weeks improved PHG-9 depression scores (-6.0 points; CI -7.9 to -4.2; *P* < 0.001) [35]. A subsequent meta-analysis of seven RCTs also noted similar improvement in depression scores [36]. Another meta-analysis, including approximately 50,000 participants, people with higher magnesium intake had one-third lower risk of depression compared to the participants who had lower magnesium consumption [37].

Magnesium’s inhibition of NMDA-mediated neuronal excitability may also suppress cortical spreading depression, an important mechanism in migraine prevention and management [38, 39]. Several trials have demonstrated that oral magnesium supplementation is beneficial for migraine prevention [40]. In the pivotal double-blind, placebo-controlled RCT by Peikert et al., trimagnesium dicitrate 600 mg daily for 12 weeks reduced migraine frequency (42% vs. 16% with placebo) [41]. Magnesium supplementation has been noted to reduce the intensity/frequency of migraine [22]. Notably, most RCTs evaluating magnesium for mood disorders and migraine prevention utilized magnesium oxide or citrate. However, in real-world clinical practice, magnesium glycinate is frequently utilized for this purpose despite its limited trial data, likely due to its improved tolerability and fewer gastrointestinal side effects [42].

### Metabolic Health and Anti-inflammatory Effects

Magnesium has been widely utilized in several aspects of improving metabolic health. Compared to the control population, hypomagnesemia is approximately 10-fold more prevalent in individuals with type 2 diabetes. Hypomagnesemia may contribute to both impaired insulin release as well as insulin resistance. Insulin resistance, in turn, reduces renal magnesium reabsorption, contributing to worsening of magnesium deficiency [43]. Insulin secretion is facilitated by the presence of magnesium, serving as a regulator of glucokinase, ATP-sensitive potassium channels, and L-type calcium channels in pancreatic β-cells [44]. Insulin resistance is mitigated by the role of magnesium as a cofactor for insulin receptor auto-phosphorylation and downstream signaling through IRS-1/2, PI3K, and protein kinase-B pathways. In addition, insulin resistance can be prevented by the indirect antioxidant effect of magnesium via its role as a cofactor for antioxidant enzymes glutathione peroxidase and superoxide dismutase [44–47]. Further, higher dietary magnesium intake is associated with a 15–22% lower risk of developing type 2 diabetes. Trial sequential analysis on RCTs and prospective cohort studies reported that each 100 mg/day increment in magnesium intake reduces diabetes risk by approximately 6% [48, 49]. Magnesium supplementation duration *≥* 4 months has been shown to enhance insulin secretion and improve insulin sensitivity. Magnesium supplementation has been shown in meta-analyses to reduce HbA1c by approximately 0.48–0.73% at doses of 500 mg/day over 24 weeks, reduce the homeostatic model assessment of insulin resistance (HOMA-IR) index by 0.41–0.67 units, and the 2-hour oral glucose tolerance test in persons at high diabetes risk [47, 48, 50–52].

Higher dietary magnesium intake has been associated with lower hypertension prevalence. Observational data suggested that each 0.5 mg/dL increase in serum magnesium has been noted with a negative association with a 7% decrease in hypertension odds [46]. Typical supplementation doses of elemental magnesium are 300–400 mg/day for 3 months. In normotensive persons, meta-analyses showed no statistical significance in reductions of systolic or diastolic blood pressure. However, significant blood pressure reductions were found to occur in specific populations such as those with higher baseline blood pressure, hypomagnesemia, or people with type 2 diabetes. For example, in patients with type 2 diabetes, magnesium supplementation of > 300 mg per day for > 12 weeks significantly reduced systolic and diastolic blood pressure by 5.78 mmHg and 2.5 mmHg, respectively. Those with hypomagnesemia had reductions of 5.97 mmHg systolic and 4.75 mmHg diastolic. Whereas, hypertensive patients on antihypertensive medication experienced the reductions in blood pressure of 7.68 mmHg systolic and 2.96 mmHg diastolic blood pressure [53–55].

Proposed mechanisms of blood pressure reduction include vasodilation through natural calcium channel blockade, increased nitric oxide production, improved endothelial function, and reduced inflammation. Magnesium deficiency activates the NLRP3 inflammasome and increases IL-1β production in antigen-presenting cells, contributing to inflammatory hypertension. In contrast, magnesium sufficiency inhibits the production of inflammatory cytokines, NF-kB, IL-6, and TNF-alpha [56–58]. Despite the data on hypertension, dietary magnesium intake has not been reliably shown to be significantly associated with cardiovascular disease or coronary heart disease. However, a strong inverse correlation was found in a meta-analysis of prospective cohort studies between magnesium intake (100 mg/day increase) and heart failure (RR: 0.69; 95% CI, 0.52–0.91; *I*^*2*^ = 0%) or stroke (RR: 0.93; 95% CI, 0.89–0.97; *I*^*2*^ = 23.8%) [59].

Observational and cross-sectional studies consistently find that lower serum or dietary magnesium was associated with higher body mass index, waist circumference, body adiposity, and obesity risk in adults and children [60]. In a 30-year cohort of more than 5,000 U.S. adults, those with the highest magnesium intake had about 50% lower obesity incidence than those with the lowest intake of magnesium [61]. Adequate magnesium intake may support healthier lipid metabolism and cardiovascular risk profiles in obesity [62–65]. Higher dietary or blood magnesium (highest category vs. lower category) was linked to a 20–40% lower risk of metabolic syndrome [66–68]. Several RCTs and meta-analyses have shown other potential beneficial effects of magnesium regarding metabolic health. For example, there were reductions in serum total cholesterol, LDL, and triglycerides. While modest increases in serum HDL have also been demonstrated [69–72]. Interestingly, co-supplementation of magnesium with vitamin D has been shown to reduce highly sensitive C-reactive protein (CRP) and tumor necrosis factor (TNF)-alpha in overweight or obese individuals, indicating potential synergistic effects with hormones or vitamins [73]. This was further reiterated in various studies where magnesium supplementation significantly reduced CRP and increased nitric oxide levels [74–76].

In a meta-analysis, Mofrad et al. evaluated the role of magnesium supplementation on vascular endothelium [77]. They investigated the effects of magnesium on flow-mediated dilatation and carotid intima media thickness as markers of endothelial function and found that magnesium supplementation improved flow-mediated dilatation but without affecting carotid intima media thickness [77]. Study heterogeneity was a significant limitation of this meta-analysis. In a cross-sectional study evaluating about 10,000 adults above 20 years noted that the prevalence of circadian syndrome (based on the presence of metabolic syndrome along with short duration of sleep and depression) was lower (35%) in people with higher magnesium intake compared to lower magnesium intake (47%) [78]. Of note, magnesium supplements have been noted to improve sleep in some studies, but the strong evidence is limited. A systematic review noted a high risk of bias and heterogeneity among the studies included [79].

### Pregnancy and Gynecological Disorders

Beyond its gastrointestinal applications, magnesium plays an important role in obstetric care and has been extensively studied for the prevention of eclamptic seizures in preeclampsia. The multinational Magpie trial demonstrated that intravenous magnesium sulfate administered after the diagnosis of preeclampsia halved the risk of eclampsia [21]. Similar to its role in migraine prevention and treatment, magnesium sulfate prevents eclamptic seizures by reducing neuronal excitability through NMDA receptor antagonism and calcium channel blockade, while promoting cerebral vasodilation. The American College of Obstetricians and Gynecologists identified magnesium sulfate as the drug of choice for seizure prophylaxis in preeclampsia with severe features and for treatment of eclampsia. Oral magnesium supplementation has been tried for the prevention of preeclampsia. In a meta-analysis, oral magnesium supplementation during pregnancy significantly reduced the risk of preeclampsia (RR: 0.76, 95% CI: 0.6 to 0.9, *P* = 0.04) [80]. However, the result was not significant for the outcome of severe preeclampsia [80]. Further, magnesium supplementation has been noted to reduce the risk of hospitalization in pregnant women [22]. Through inhibition of prostaglandin synthesis and relaxation of uterine smooth muscle, RCTs also suggest that magnesium supplementation can reduce pain severity in primary dysmenorrhea, with oral supplementation associated with reductions in menstrual pain severity and associated symptoms compared to placebo [81].

### Asthma and Other Pulmonary Disorders

Magnesium has been used in the treatment of asthma exacerbations since the 1930s due to its calcium channel–blocking effects on bronchial smooth muscle, resulting in bronchodilation [5, 82]. The Global Initiative for Asthma (GINA) currently recommends intravenous magnesium sulfate for severe, refractory asthma exacerbations that do not respond to initial therapies. As of the 2025 published GINA guidelines, nebulized magnesium is no longer recommended for asthma exacerbations in children, adolescents, or adults due to a lack of significant benefit [83]. This context-specific clinical recommendation has at times been generalized to support oral magnesium supplementation for chronic asthma management. However, clinical evidence supporting this practice is lacking [84]. A 2019 meta-analysis of 917 asthmatic patients found no consistent improvement with oral magnesium supplementation, apart from an isolated improvement in forced expiratory volume in one second (FEV1) at eight weeks that was not observed at other time points [85]. External validity of these findings is limited by considerable heterogeneity among the studies included in the analysis. A recent Cochrane database systematic review did not find any evidence to support the use of magnesium sulphate for managing acute bronchiolitis in children less than two years of age [86].

### Bone Disorders

Studies have noted that reduced bone density with higher incidence of osteoporosis and fractures have been associated with low dietary magnesium intake and low serum magnesium levels [87–90]. A meta-analysis demonstrated a significant positive correlation between magnesium intake and hip bone mineral density [7].

### Cognitive Function

Since the 1990s, magnesium has been suggested to play a role in Alzheimer’s dementia after its relative insufficiency was identified in the brain [91]. Magnesium crosses the blood–brain barrier and has been proposed to facilitate neuronal maturation, toxin clearance, attenuate neuroinflammation, ameliorate pathological precursors of amyloid and abnormal tau phosphorylation, and reverse dysregulation of NMDA receptors [92]. However, the exact mechanisms underlying these processes have not been fully elucidated [93]. In experimental models, magnesium L-threonate has been reported to decrease neuroinflammation and beta-amyloid deposition [94, 95]. In rats, magnesium L-threonate has been shown to improve learning capabilities as well as both short-term and long-term memories [96].

One epidemiologic study of 1,400 adult men found an association between increased dietary magnesium intake and a reduced risk of mild cognitive impairment over an eight-year period [97]. Another epidemiologic study in Japanese participants over sixty years of age found decreased odds of dementia and vascular dementia among those consuming > 200 mg/day of magnesium over a seventeen-year period [98]. However, more rigorous studies are needed to support associations between magnesium and cognitive health. A 2024 meta-analysis examining this relationship screened 3,812 articles and identified only twelve cohort studies and three RCTs suitable for analysis. The authors noted considerable heterogeneity among cohort studies and an insufficient number of RCTs to draw firm conclusions about magnesium supplementation [99].

Magnesium deficiency has also been implicated in the pathogenesis of Parkinson’s disease. One meta-analysis found significantly lower levels of magnesium in cerebrospinal fluid among individuals with Parkinson’s disease compared to controls [100]. The authors noted that most included studies were of poor quality. Importantly, association does not imply causation. While magnesium has been linked to several neuroprotective mechanisms, including NMDA receptor inhibition and reduction of oxidative stress, additional rigorous studies are required to determine any potential therapeutic benefit [93, 100].

Similar to Parkinson’s disease and Alzheimer’s dementia, magnesium has been implicated in attention deficit hyperactivity disorder (ADHD), primarily through modulation of dysregulated NMDA receptors [101]. A meta-analysis of seven observational studies found that patients with ADHD had significantly lower serum magnesium levels compared to controls; however, significant heterogeneity was observed among study populations. Although this suggests a possible association between magnesium levels and ADHD, the clinical efficacy of magnesium supplementation has not been definitively demonstrated. For example, one RCT of 135 pediatric patients found no improvement in ADHD scores between treatment arms [102]. A smaller RCT of sixty-six pediatric patients suggested behavioral improvements when magnesium was combined with vitamin D supplementation [103], though generalizability is limited by the small sample size. Overall, robust data supporting therapeutic magnesium supplementation for ADHD are lacking, and current guidelines do not recommend magnesium in the management of ADHD [104].

### Muscle Function and Athletic Performance

Magnesium is a common component of pre-workout drinks, electrolyte rehydration solutions, and other products aimed at optimizing muscle function, exercise performance, and recovery. Magnesium supplementation has been hypothesized to modulate glucose metabolism and reduce inflammation, thereby enhancing exercise performance. A small, double blind, between-group study evaluated muscle soreness and performance before and after supplementation with 350 mg of magnesium glycinate [105]. Although limited by the subjective nature of outcomes, participants reported significantly reduced muscle soreness, lower session and acute ratings of perceived exertion, and improved perceived recovery after magnesium (vs. placebo) supplementation, with some evidence suggesting a positive impact on performance.

### Aging

Some of the cardinal hallmarks of aging include genomic instability, telomere attrition, epigenetic modifications, mitochondrial dysfunction, loss of proteostasis, dysregulated nutrient sensing, and stem cell exhaustion [106]. Increased inflammation, impaired autophagy, and dysbiosis have also been proposed as additional hallmarks. Magnesium has been mechanistically linked to several of these processes, including mitochondrial dysfunction, telomere length regulation, genomic instability, and loss of proteostasis [107]. However, clinically meaningful endpoints are lacking in this area of research, and larger, well-designed studies are needed to substantiate these associations. For example, one small RCT of magnesium L-threonate demonstrated possible improvements in overall cognitive ability, but the study duration was only 12 weeks, and the sample size was limited [98]. Such short-term studies are incongruent with long-term endpoints such as lifespan and aging, which magnesium is purported to influence.

### Miscellaneous Disorders

A systematic review evaluated the role of magnesium in chronic pain disorders in adults, and studies included patients with complex regional pain syndrome and back pain [108]. The efficacy of magnesium in reducing chronic pain disorders from these studies was equivocal, recommending further exploration of the role of magnesium [108]. The exact role of magnesium in many conditions, such as sleep disorders, muscle cramps, and restless leg syndrome, remains elusive, and available high-quality evidence remains limited [79, 109–111]. Observational research noted a positive correlation between magnesium and sleep quality, while the RCTs reported contradictory findings. Well-designed randomized clinical trials with a larger sample size and longer follow-up time (more than 12 weeks) to further clarify the relationship are urgently needed.

## Current Challenges and Future Directions

The lack of an accurate biomarker for the estimation of magnesium status limits our interpretation in many clinical trials. Experts currently recommend a combination of serum magnesium, 24-hour urinary magnesium, and dietary magnesium intake as the best way to determine magnesium status, rather than using serum levels alone, and this is not pragmatic and very cumbersome. Further, the availability of various forms of magnesium supplements (different strengths and formulations) and their bioavailability is challenging to generalize the conclusions of that particular preparation. Also, more long-term data is currently needed, as most published studies are either observational in nature or short-term interventional trials.

## Conclusions

Low magnesium can be noted in many clinical scenarios, such as decreased magnesium intake, reduced absorption, and increased excretion, and should be actively evaluated and managed. Multiple studies noted the significant role of magnesium in the management of many conditions, such as eclampsia, asthma, migraine, chronic gastrointestinal disorders, metabolic dysfunction (diabetes, hypertension, dyslipidemia, and obesity), and mood disorders. On the contrary, many products are also sold over the counter solely based on the purported benefit of magnesium compounds, based on their physiological role in many metabolic pathways. The upper intake level for magnesium from supplemental products is 350 mg for adults, and for the pediatric population, it ranges from 65 to 350 mg (based on the age of children and adolescents). Very large doses of magnesium supplements, even in healthy populations, can lead to hypermagnesemia, which can lead to life-threatening manifestations. As systemic magnesium is predominantly eliminated via the kidneys, routine administration of these supplements in patients with renal insufficiency should be avoided to prevent hypermagnesemia. Further, population-based rigorous studies are urgently needed to evaluate the benefits and side effects of routine magnesium supplementation.

## Key References

- Touyz RM, de Baaij JH, Hoenderop JG. Magnesium disorders. New England Journal of Medicine. 2024;390(21):1998-2009.
  - ○ This article comprehensively addresses the role of magnesisum in humans and details the magnesium disorders.
- Freedman MR, Fulgoni VL, Lieberman HR. Temporal changes in micronutrient intake among United States Adults, NHANES 2003 through 2018: A cross-sectional study. The American Journal of Clinical Nutrition. 2024;119(5):1309-20.
  - ○ In this NHANES article, changes in magnesium intake from 2003 to 2018 was detailed.
- Moabedi M, Aliakbari M, Erfanian S, Milajerdi A. Magnesium supplementation beneficially affects depression in adults with depressive disorder: a systematic review and meta-analysis of randomized clinical trials. Frontiers in psychiatry. 2023;14:1333261.
  - ○ This article is an important systematic review and meta-analysis that comprehensively addressing the benefits of mangnesium supplemenation in depressive disorder.
- Talandashti MK, Shahinfar H, Delgarm P, Jazayeri S. Effects of selected dietary supplements on migraine prophylaxis: a systematic review and dose–response meta-analysis of randomized controlled trials. Neurological sciences. 2025;46(2):651-70.
  - ○ This systematic review and meta-analysis details the effeet of dietary supplmentnts on migraine prophylaxis.
- Tirani SA, Rouhani P, Saneei P. Hypertension in relation to circulating magnesium levels: a systematic review and meta-analysis of observational studies. Nutrition reviews. 2025;83(7):1277-89.
  - ○ This systematic review and meta-analysis evaluted the effects of magnesium levels on hypertension.

## Data Availability

No datasets were generated or analysed during the current study.
